# [Retracted] MicroRNA-298 inhibits malignant phenotypes of epithelial ovarian cancer by regulating the expression of EZH2

**DOI:** 10.3892/ol.2026.15493

**Published:** 2026-02-16

**Authors:** Fenmei Zhou, Juan Chen, Hairong Wang

Oncol Lett 12: 3926–3932, 2026; DOI: 10.3892/ol.2016.5204

Following the publication of the above paper, it was drawn to the Editor's attention by a concerned reader that the ‘miR-298 mimics’ data panel for OVCAR3 cells in Fig. 4B on p. 3929 was very similar to data that had already been published in a paper in the journal *PLoS One* by different authors at different research institutes. In addition, the scratch-wound assays shown in Fig. 3 showed some evidence of image duplications; although not entire duplications of the data panels, several overlapping features comparing among various of the panels were identified. Finally, upon performing an independent analysis of the data in this paper in the Editorial Office, it also came to light that the cellular images in Fig. 4 contained apparent anomalies, namely the repeated patternings of small areas of cellular data within the same figure panels.

Owing to the fact that the contentious data in the above article had already apparently been published prior to its submission to *Oncology Letters*, in addition to the potentially anomalous presentation of data in Fig. 4, the Editor has decided that this paper should be retracted from the Journal. The authors were asked for an explanation to account for these concerns, but the Editorial Office did not receive a reply. The Editor apologizes to the readership for any inconvenience caused.

